# The Use of Atomic Force Microscopy for 3D Analysis of Nucleic Acid Hybridization on Microarrays

**Published:** 2015

**Authors:** E. V. Dubrovin, G. V. Presnova, M. Yu. Rubtsova, A. M. Egorov, V. G. Grigorenko, I. V. Yaminsky

**Affiliations:** Department of Physics, Lomonosov Moscow State University, Leninskie gory, 1/2, 119991, Moscow, Russia; Department of Chemistry, Lomonosov Moscow State University, Leninskie gory, 1/3, 119991, Moscow, Russia; Russian Medical Academy of Postgraduate Education, Barrikadnaya, 2/1, 125993, Moscow, Russia

**Keywords:** DNA, oligonucleotide microarrays, hybridization, atomic force microscopy, gold nanoparticles, CTXM type β-lactamases

## Abstract

Oligonucleotide microarrays are considered today to be one of the most efficient methods of gene diagnostics. The capability of atomic force microscopy (AFM) to characterize the three-dimensional morphology of single molecules on a surface allows one to use it as an effective tool for the 3D analysis of a microarray for the detection of nucleic acids. The high resolution of AFM offers ways to decrease the detection threshold of target DNA and increase the signal-to-noise ratio. In this work, we suggest an approach to the evaluation of the results of hybridization of gold nanoparticle-labeled nucleic acids on silicon microarrays based on an AFM analysis of the surface both in air and in liquid which takes into account of their three-dimensional structure. We suggest a quantitative measure of the hybridization results which is based on the fraction of the surface area occupied by the nanoparticles.

## INTRODUCTION

The oligonucleotide microarray technology is a relatively new method which appeared in the mid-1990s and is based on hybridization of oligonucleotide probes with target nucleic acids [1]. This method allows a simultaneous analysis of a large number of nucleic acids sequences and is a powerful tool for clinical diagnosis [2], assessment of drug sensitivity [3], and toxicological studies [4]. It is also used in other scientific and practical fields of biology and medicine [5].

A DNA microarray is composed of a solid support with a large number of immobilized oligonucleotide probes with known sequences. These probes are capable of hybridizing with the complementary DNA or RNA fragments from a test sample. The use of fluorescent dyes is the most common method to detect the result of hybridization of the probes with the target DNA [6]. Radioisotopes [7], enzymes [8], and gold nanoparticles [9] are also used as labels in the microarray technology. Along with optical and fluorescence detection, electrochemical detection [10] and surface plasmon resonance [11] are used. Over recent years, the microarray surface has extensively been studied by high-resolution microscopy. Scanning electron microscopy (SEM) was used to examine the surface of glass microarrays with biotin-labeled oligonucleotide probes that were detected using streptavidin-peroxidase polymers and silver reduction enhancement [12]. It was shown that the silver nanoparticles formed during amplification are adsorbed on the surface and are clearly distinguishable on the surface using this method. Scanning electron microscopy was used to record sandwich hybridization of a model single-stranded DNA composed of 46 nucleotides. For that purpose, first-type oligonucleotide probes were immobilized on a support and DNA was detected using second-type oligonucleotide probes ; labeled with gold nanoparticles [13]. A simple method of counting the number of particles per unit area was suggested, which provided high sensitivity and a better signal-to-noise ratio compared to those obtained with a fluorescent probe.

Atomic force microscopy (AFM), which is based on the operating principle of a profilometer, an instrument used to measure surface irregularities, has approximately the same lateral resolution as SEM, but considerably surpasses SEM in vertical resolution. Furthermore, AFM does not require a vacuum environment for sample examination and, thus, allows one to study samples under various conditions both in air and in liquid.

It should be noted that atomic force microscopy has gained considerable currency in the analysis of adsorbed DNA and RNA molecules without the use of labels [14-16]. A number of studies have used AFM as a tool for imaging and analyzing biospecific interactions, such as binding of bacterial cell fragments to antibodies in solution [17], binding of bacteriophages to the host cell [18], and other receptor-ligand interactions [19]. The use of AFM to analyze the surface topography of DNA microarrays has been reported. It facilitated the optimization of their preparation technology [20, 21]. The advantages of this method include the fact that there is no need for special preparation of the microarray surface and the relatively simple connection of a microarray to a microscope for further analysis (e.g., in most cases, one has to simply attach a support to a special magnetic disk). AFM analysis of the surfaces of DNA microarrays after their exposure to a sample solution led to a conclusion about hybridization of the probes with the complementary target DNAs [22] or gold nanoparticles incorporated therein [23]. In a number of studies, AFM made possible the development of quantitative criteria for the evaluation of target DNA hybridization on a microarray surface. A quantitative approach to an AFM analysis of DNA microarrays is extremely important, since it allows a quantitative comparison of the hybridization efficiency, in particular under significantly lower (compared to conventional detection methods) concentrations of the target. For example, the layer height on the biochip surface evaluated using AFM-nanolithography was used as a quantitative criterion [24]. In the cases where hybridized targets are morphologically distinguishable on the surface, the amount of bound DNA targets [25] or the nanoparticles associated with them [26] per unit surface area may serve this criterion. In this work, we developed an AFM-based approach to study silicon oligonucleotide microarray surfaces after hybridization, with the possibility of a quantitative analysis of its results. Allowance for the total area occupied by the targets (the nanoparticles associated with them) bound to the microarray surface is a special feature of the developed approach.

We assumed that the unique capability of AFM to visualize single targets (nanoparticles) on the microarray surface and provide information on their height and other sizes will provide an additional morphology- based criterion for the selection of “true” targets and, thus, lower the threshold for the detection of targets, increase the signal-to-noise ratio, and also reduce the amount of material required to produce microarrays. Nucleic acids encoding bacterial CTX-M type β-lactamases, which are responsible for the development of resistance to cephalosporins in Gram-negative bacteria (causative agents of infectious diseases), were used as model DNAs [27, 28].

## EXPERIMENTAL

Gold nanoparticles were prepared according to the Frens method based on the reduction of chloroauric acid with sodium citrate [29]. The size of the gold nanoparticles was assessed by SEM using a Supra-40 scanning electron microscope (Carl Zeiss, Germany) equipped with an InLens secondary electron detector built in the microscope column.

Streptavidin (2 mg in 200 µl of 10 mM K-phosphate buffer, pH 7.2) was modified with 3.2 mg of mercaptosuccinic acid in the presence of 3 mg of carbodiimide at +4°C overnight to obtain streptavidin conjugated to gold nanoparticles. Thereafter, 10 µl of 10 mM EDTA was added, the mixture was stirred at room temperature for 30 min, and the resulting solution was dialyzed against phosphate buffer with EDTA. The colloidal gold solution pH was adjusted to 7.0 using a freshly prepared Na_2_CO_3_ solution, and then streptavidin modified with mercaptosuccinic acid was added. After incubation at room temperature for 1 h, the solution was centrifuged (30 min, 11,000 rpm, 4°C). The supernatant was then removed, and the precipitate was dissolved in 10 mM K-phosphate buffer with pH 7.2.

The 5’-TTTTTTTTTTTTTT-ATATCGCGGTGATCTGGCC- 3’ probe was used to identify nucleic acids encoding CTX-M type β-lactamases. The 5’-TTTTTTTTTTTTTT-CTAGACAGCCACTCATA-3’ probe was used to control non-specific hybridization. These probes were modified with an amino group at the 5’-end.

Amplification of CTX-M type β-lactamase genes of 870 bp with simultaneous inclusion of biotin was performed by PCR as described in [30].

The surface of silicon plates was purified with oxygen plasma using a RDE-300 reactive ion-etching instrument (Alcatel, France) for 30 min. Then, it was chemically modified [31]: silicon was treated with a 10 mM solution of 3-glycidyloxypropyl trimethoxysilane (GPTMS) in dry toluene at 80°C for 12 h then washed and heated at 100°C for 10 min. The surface of the modified silicon was covered by oligonucleotide probes with the 5’-end amino group using 20 pmol/μl solutions in 0.25 M Na-phosphate buffer containing 0.3 M Na_2_SO_4_. After immobilization, free protein binding sites on the silicon surface were blocked in a solution of 1% BSA and 1% casein in 10 mM K-phosphate buffer, pH 7.2, containing 0.15 M NaCl. Hybridization of 1 nM of biotin-labeled DNA was performed on an oligonucleotide microarray in buffer containing 0.05 M NaH_2_PO_4_, 0.5 M NaCl, and 0.005 M EDTA (pH 7.4) at a temperature of 45°C for 2 h. Washing was performed with 10 mM K-phosphate buffer (pH 7.2) containing 0.15 M NaCl and 0.1% Tween 20. The microarray was then incubated with a solution of streptavidin conjugated with gold nanoparticles with a protein concentration of 40 ng/ml at 37°C for 45 min and then washed.

In this work, we used a Nanoscope IIIa atomic force microscope (Digital Instruments, USA) in the tapping mode. Scanning was performed in air using fpN10 commercial cantilevers (Mikromash, Estonia) and in liquid using NP-S1 cantilevers (Veeco, USA) with a scanning frequency of 2.1 Hz, 512 × 512 dots. Processing and analysis of images were performed using the Femto- Scan Online software (Advanced Technologies Center, Russia).

## RESULTS AND DISCUSSION

The hybridization analysis on silicon microarrays was performed using oligonucleotide probes immobilized on a silicon surface modified with γ-glycidyloxypropyl trimethoxysilane (GPTMS). The structure of the oligonucleotide probe used to detect nucleic acids encoding CTX-M type β-lactamases and the structure of the control probe are provided in the Experimental section. The hybridization reaction was carried out using a target DNA of 870 bp to which biotin molecules were incorporated during PCR. Biotin molecules in duplexes formed on the surface were detected using streptavidin conjugated with gold nanoparticles. We used spherical gold nanoparticles with a size of 27 ± 3 nm. After hybridization and detection of the duplexes using streptavidin conjugated with gold nanoparticles, the microarray surface was examined by AFM.

**Fig. 1:**
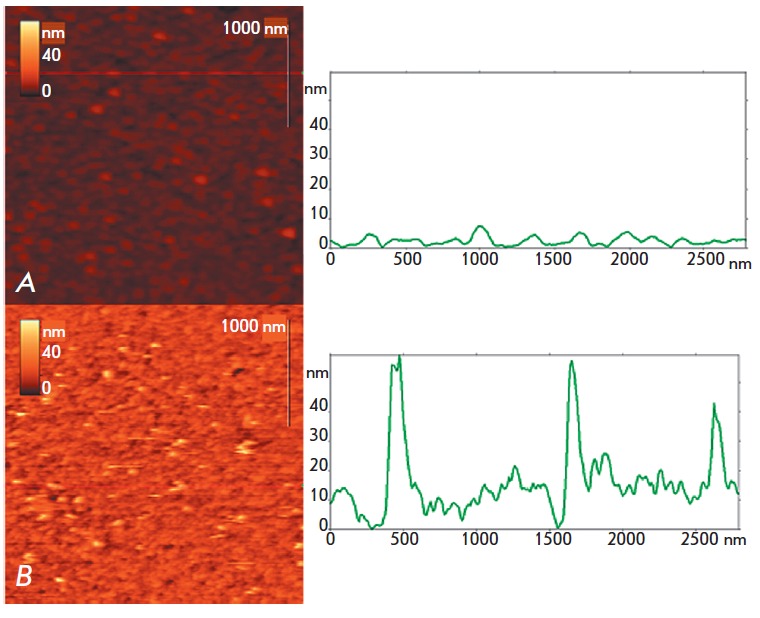
AFM images of microarray surfaces obtained in buffer before (A) and after (B) hybridization with the biotin labeled target DNA and interaction with streptavidin conjugated to gold nanoparticles. On the right, vertical profiles of the microarray surfaces are shown along the line drawn in the corresponding image on the left

*Fig. 1* shows the AFM images of the microarray surface obtained in a buffer before and after hybridization with gold nanoparticle-labeled nucleic acids encoding CTX-M-3 β-lactamases. The microarray surface with DNA duplexes is morphologically composed of tightly packed globules 5–10 nm in diameter that consist of silicon modified by γ-GPTMS and oligonucleotides. Similar structures were previously observed in the case of other oligonucleotide microarrays on silicon [22]. After hybridization with labeled DNA, nanoparticles, which are markers of hybridization products, appear on this surface. Their height is 30–50 nm *(Fig. 1B). *Images of the nanoparticles in a liquid medium are unstable, blurred, and replete with numerous scan failures, which is manifested in the appearance of light bands. This is likely due to weak fixation of DNAbound nanoparticles on the surface, since only a small portion of the nucleic acid (18 nucleotides of 870) is involved in hybridization. Due to the weak adhesion of nanoparticles to the surface, the nanoparticle height measured by AFM exceeded the value of 27 ± 3 nm obtained for these particles by SEM. Detection of gold nanoparticles, which are part of DNA duplexes, on the microarray surface is an important result, as it proves* in situ *binding of oligonucleotides to complementary DNA sites. It is advisable to perform a quantitative evaluation of DNA hybridization results after drying the microarray surface, to increase the stability of AFM images of gold nanoparticles.

**Fig. 2:**
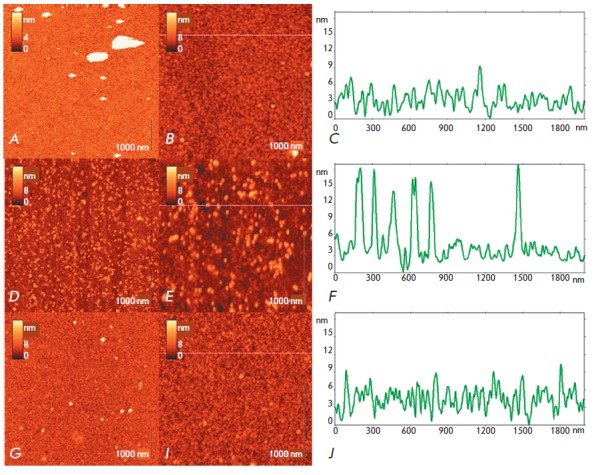
AFM images and vertical profiles of the silicon microarray surface: (A–C) – immobilized oligonucleotide probes on γ-GPTMS modified silicon before exposure to an analyzed solution, (D–F) – after exposure to the analyzed solution of the target DNA and incorporation of nanoparticles into the duplexes, (G–I) – microarray areas without oligonucleotide probes on the surface, after their exposure to the analyzed solution of the target DNA and streptavidin conjugated to gold nanoparticles

Typical AFM images and the oligonucleotide microarray surface profile prior to hybridization, which were obtained in air, are shown in *Fig. 2A-C*. In this case, the microarray has a uniform surface consisting of globules of up to 10 nm in height, on which there are randomly shaped objects up to 330 nm in height (white structures in *Fig. 2A*). The globular surface in general reproduces the surface pattern observed in buffer *(Fig. 1A)*. In this case, the high objects are probably impurities from buffer solutions and contaminants from the air, and they appear randomly during microarray preparation and DNA identification. AFM allows direct control of the total area of these structures, which is small compared to the microarray working surface.

Microarray surface images with DNA duplexes labeled with gold nanoparticles are shown in *Fig. 2 D-F*. They demonstrate a large number of individual spherical particles 10–30 nm in height and their small aggregates composed of 10–15 particles. With allowance for the diameter of the used gold nanoparticles (27 ± 3 nm) and the possibility of their partial immersion into the oligonucleotide matrix during the hybridization of probes with target DNAs, the spherical particles observed in AFM images may be interpreted as gold nanoparticles, which are markers of hybridized DNA molecules.

*Fig. 2G* –*I *shows AFM images and the surface profile of the microarray control region not covered with oligonucleotide probes, with hybridization with the DNA target followed by incorporation of gold nanoparticles being performed by the standard procedure, to control the hybridization specificity. In these images, a small amount (compared to *Fig. 2 D-F*) of differently sized objects is observed on the background of γ-GPTMS modified silicon.

In order to interpret the results of the hybridization of probes with targets containing gold nanoparticles as labels, we developed a method for the quantitative analysis of AFM images of the microarray surface. It is based on the 3D-analysis of the microarray surface, i.e. on allowance for the heights and areas of the objects detected on the substrate surface after completion of all stages of the analysis. AFM provides information on the object’s height with a high accuracy of up to a tenth of a nanometer, which allows one to range the objects observed on the microarray surface according to their height. Thus, it becomes possible to detect the results of complementary hybridization based on the height of the nanoparticles used as labels. In this case, we can disregard the objects non-specifically bound to the microarray surface, which have a smaller height.

**Fig. 3:**
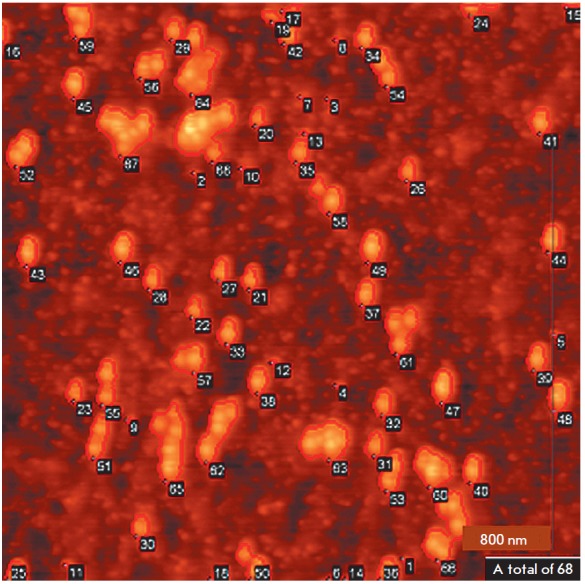
Algorithm for object selection in the AFM image. Selected objects are outlined

All of the observed objects were selected for the analysis of the AFM images shown in *Fig. 2 A,D,G*. The mathematical algorithm of this selection was a search for the zero background level in a histogram as the most probable height distribution of all 512 × 512 dots in an AFM image and construction of the threshold plane above which all parts of the surface were taken as objects. This algorithm is integrated into the semi-automatic function of the software used for image processing (see the method). All selected objects are characterized by a number of easily computed geometric characteristics, such as the height, area, volume, perimeter, form factor reflecting the object shape, etc. *Fig. 3* shows an example of automatic selection of objects in an AFM image containing gold nanoparticles.

The height range, within which the selected objects will be considered as labels, is selected individually in each task based on information on the used labels and the structural features of the microarray. We used the values 10 and 30 nm as the lower and upper limits of this filter. Selection of the upper limit (30 nm) was related to the known gold nanoparticle diameter distribution of 27 ± 3 nm obtained by SEM. Due to the fact that objects higher than 30 nm were rarely observed in AFM images of microarrays after hybridization with DNA targets labeled with gold nanoparticles, we did not consider the possibility of a “vertical” arrangement of nanoparticle aggregates on the surface. Since the AFM-measured object height may be underestimated (due to surface deformation by cantilever) and also taking into account the possible partial immersion of a gold nanoparticle into the oligonucleotide (and GPTMS) matrix, the lower limit (10 nm) of the range was selected empirically based on the analysis of the lower limit of the gold nanoparticle height distribution in the corresponding AFM image. In principle, an algorithm can be developed for this step (selection of the height range filter) by selecting a threshold value for the fraction of objects observed within a given range with respect to the total number of objects observed on the surface. On surfaces with a small amount of impurities, this threshold value will be close to one; i.e., most of the observed objects will represent nanoparticles (the selected range corresponds to a threshold value of 0.9).

**Fig. 4:**
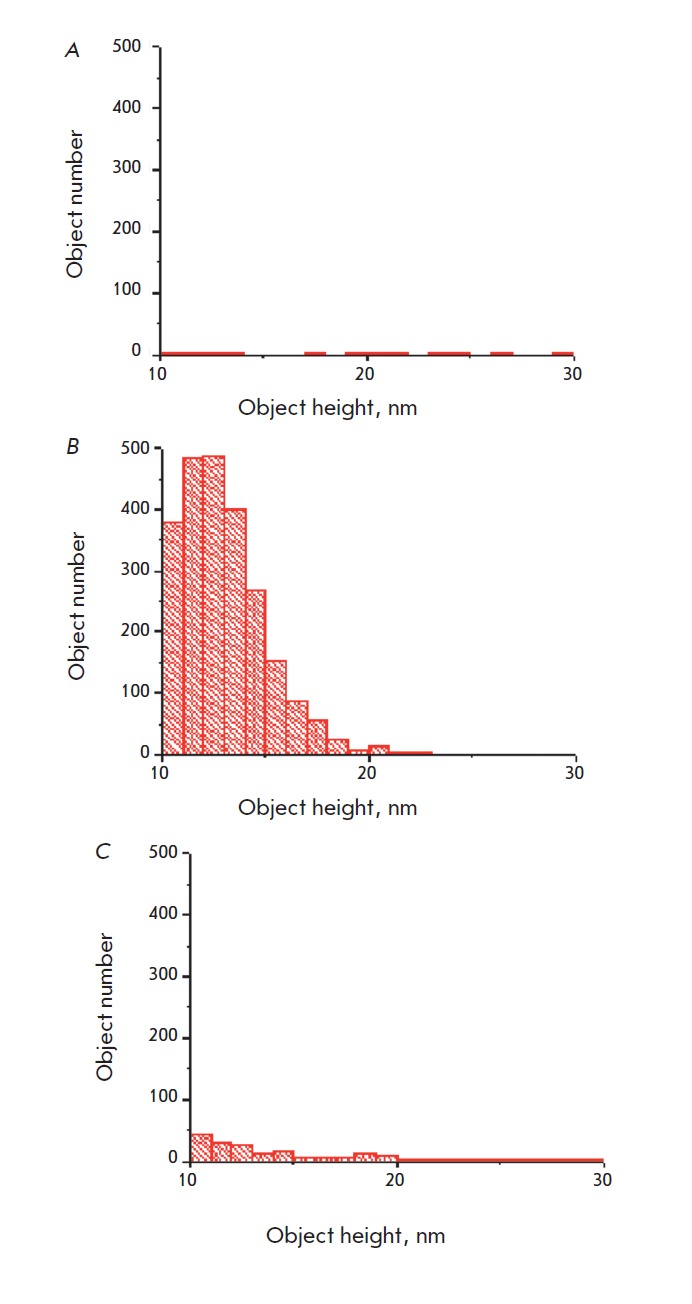
Histograms for objects height distribution in the range of 10–30 nm observed in AFM images of the microarray surface before (A) and after (B) hybridization with the target DNA and streptavidin conjugated to gold nanoparticles and also for the control surface without oligonucleotide probes after exposure to the analyzed DNA target solution (C). Data for each histogram are summarized over three AFM images sized 5 × 5 μm

**Fig. 5:**
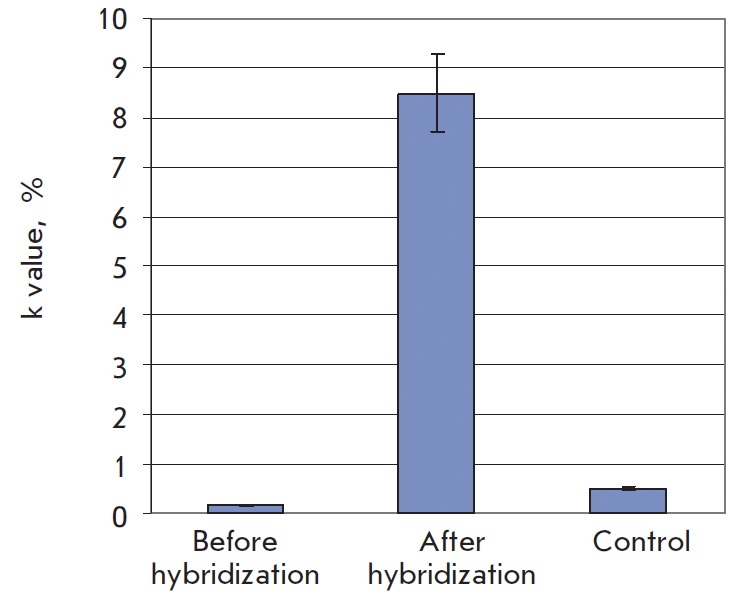
*k *value (in %) for the microarray surface before and after hybridization with the target DNA and streptavidin conjugated to gold nanoparticles and also for the control surface without oligonucleotide probes after exposure to the analyzed DNA target solution. The parameter was calculated based on three AFM images sized 5 × 5 μm

Histograms of the object’s height distribution in the selected range are shown in *Fig. 4* for the microarray working surface prior to hybridization *(Fig. 4A)*, after hybridization *(Fig. 4B), *and also for the control surface of microarrays without immobilized probes, which is exposed to a solution with the target DNA under the same experimental conditions (*Fig. 4C*). The histograms summarize data obtained from AFM images in three different fragments of each surface. For clarity, the histograms are shown on the same scale. The total area of the objects, which were selected based on their heights *(si)*, normalized to the total area of the AFM image *Si:*:*k *= Σsi/S and expressed as a percentage was used for a quantitative comparison of the hybridization efficiency. In this way, accounting of nanoparticle aggregates will be more effective, because their area is proportional to the number of aggregated particles. The parameter *k *reflects the fraction of the area occupied by nanoparticle labels with respect to the total microarray surface. In connection with the effect of broadening protruding objects by a cantilever of an atomic force microscope, it should be borne in mind that the *k *parameter is an upper estimate for the fraction of the area occupied by nanoparticles. *Fig. 5* shows *k* values and their related errors for the experiments. The fraction of the area occupied by gold nanoparticles upon complementary hybridization was estimated to be 8%; in the absence of complementary binding –0.5%, whereas the background particle surface area did not exceed 0.2%. In this case, the signal-to-noise ratio was 16 and 40, respectively. For reference, the signal-tonoise ratio for fluorescence detection was 10 (for a target concentration of 1 nM) [13].

Our approach allows the use of a quantitative criterion to assess the hybridization of oligonucleotide probes with target DNA and makes it possible to compare the efficiency of DNA identification on various microarrays. An important difference between our approach and conventional methods for detecting hybridization of a probe with the target DNA, as described in Introduction (e.g., fluorescence and optical detection), is the possibility to visualize single target binding events. Due to this fact, the detection threshold for target DNAs can be significantly reduced compared to conventional methods, which require the presence of simultaneous signals from a large number of bound targets. For example, in reference [13], where the hybridization efficiency was assessed by direct counting of nanoparticles in SEM images, the minimum detection threshold was achieved by detection of one nanoparticle per square micrometer, on average, with the minimum detectable concentration being 1,000 times lower compared to that for detection using fluorescent labels.

It should be emphasized that the use of our approach (and the *k *parameter*) *is not limited to systems using nanoparticles as DNA markers. This approach can be applied to the detection of molecules or other targets without the use of labels. In this case, the *k *parameter (or its equivalent, where the numerator is the sum of volumes rather than areas) will characterize the amount of bound material (target).

## CONCLUSIONS

In this work, the AFM method was used to study oligonucleotide microarrays for the identification of DNAs encoding bacterial CTX-M type β-lactamases. Incorporation of gold nanoparticles into DNA duplexes allows the use of AFM for effective detection of the hybridization of target DNA with oligonucleotide probes both in air and in liquid. In order to quantify the nucleic acid hybridization processes, we developed an approach to evaluate the results of hybridization using a three-dimensional analysis of AFM images of the microarray surface, which accounts for the height and area of the gold nanoparticles used as labels. This method allows one to ignore particles that are non-specifically bound to the surface and differ from labels in height, as well as to take into account aggregates of target nanoparticles, which increases the detection efficiency. In the case of the silicon microarrays studied in this work, the parameter *k, *corresponding to the fraction of the area occupied by nanoparticles after hybridization with labeled specific DNA, was equal to 8%, while the control values did not exceed 0.5%. The main advantage of AFM over other methods for detection of binding on oligonucleotide microarrays is its capability to gain the three-dimensional morphology of individual hybridized DNA molecules. The obtained information on the three-dimensional structure of an object allows the use of more accurate morphological criteria for the detection of hybridized DNA molecules.

## Abbreviations

SEM: scanning electron microscopy
AFM: atomic force microscopy
EDTA: ethylenediaminetetraacetic acid
GPTMS: γ-glycidyloxypropyl trimethoxysilane
PCR: polymerase chain reaction
